# The Difficult Alliance between Vegan Parents and Pediatrician: A Case Report

**DOI:** 10.3390/ijerph17176380

**Published:** 2020-09-02

**Authors:** Ilaria Farella, Raffaella Panza, Maria Elisabetta Baldassarre

**Affiliations:** 1 Departement of Pediatrics, Queen Fabiola Children’s University Hospital, 1020 Bruxelles, Belgium; ilafarella@yahoo.com; 2 Departement of Biomedical Science and Human Oncology, Neonatology and Neonatal Intensive Care Unit, «Aldo Moro» University of Bari, 70100 Bari, Italy; mariaelisabetta.baldassarre@uniba.it

**Keywords:** vegetarianism, children, failure to thrive

## Abstract

The number of children on a vegetarian or vegan diet is gradually increasing. If not balanced and adequately supplemented, these dietary regimes can seriously impact the growth of children. Often the pediatrician is not perceived as a figure to rely on in the event of parents’ willingness to follow an alternative diet for their child. The feeling of distrust of parents towards the pediatrician can be dangerous for the health of the child. We present a 22-month-old boy with failure to thrive probably induced by an unbalanced vegetarian diet. The acquisition of the anamnestic data concerning the child’s diet was difficult because initially omitted by the parents. The poor compliance and the difficult follow-up highlights the difficulty in establishing a therapeutic alliance between parents who follow alternative regimens and the pediatrician.

## 1. Introduction

Failure to thrive (FTT) has several causes in children that need to be carefully investigated. The most common cause of FTT is the inadequate caloric intake. Other causes are: inadequate nutrient absorption, increased metabolism or a combination of these mechanisms [1]. It is important to emphasize that FTT is due to an organic pathology in only 1.4% of cases. [2]. A systematic approach based on a detailed history, physical examination and growth parameters longitudinally evaluated is therefore essential to make a correct diagnosis, avoiding invasive tests. A dietary history that investigates in particular the fact of following alternative regimes (e.g., vegan or vegetarian) is fundamental as these diet regimes have become increasingly popular [3]. Alternative diets, in fact, if not carefully followed and planned, can be counted among the causes of failure to thrive as well as severe nutritional deficiencies [4,5,6]. It is therefore important to intercept families who have this desire for their children and try to better balance the diet to prevent dangerous effects on the health of the young patients.

## 2. Case Report

A 22-month-old boy came to our attention for failure to thrive. Since the age of 15 months the parents have consulted three different pediatricians and a pediatric gastroenterologist. The mother reported that she followed varied diet during last pregnancy. She had followed a vegan diet during a previous pregnancy, suspended for iron deficiency anemia onset. The baby was born at term showing a birth weight of 3460 g (56th centile), a length of 50 cm (42nd centile), an head circumference of 35 cm (61st centile); perinatal course was uneventful. He was exclusively breastfed for 10 months and then he received a rice-based drink (formulation not suitable for infants). The parents reported that the child followed an omnivorous diet but that he preferred carbohydrates to proteins. Based on the alimentary recall diary of the previous three days we found out that the child did not have meat, eggs, or dairy. The parents stated that they were not in favour of the use of these alimentary products, however they allowed the child to have fish in a small quantities (weekly). The food intake in quantity and frequency was satisfactory: he had three main meals and two snacks. Neither swallowing issues nor coughing or vomiting during meals were reported. He had a regular intestinal transit. Psycho-motor development was normal. Vaccinations were partially carried out by choice of parents. Neither previous hospitalizations, nor surgery have been previously reported. Positive family history of celiac disease is reported in the maternal branch (grandfather). Laboratory complementary tests prescribed by the pediatric gastroenterologist showed: normal blood count, urine, liver function, calcium, phosphorus and alkaline phosphatase; decreased iron level (17 mcg/dL) [n.r. 30–130] and ferritin (10.12 ng/dL) [n.r.18, 2–341] and low total IgA (0.25 g/L) [n.r.0.8–4.5]. Celiac disease assessment revealed the presence of haplotype DR4-DQ8 (that is associated with an increased risk of celiac disease), but negativity of anti-endomysial antibody and Anti-transglutaminase IgG and IgA. Abdominal ultrasound was normal. The pediatric gastroenterologist recommended an esophagogastroduodenoscopy to rule out coeliac disease. During the visit, the physical examination was normal except for the presence of skin pallor and slightly flecked eyes. Weight: 10 kg (3th), height: 81 cm (14th), head circumference (26th). The patient received oral iron supplements for about a week as prescribed by the pediatric gastroenterologist, but the mother did not respect the recommended diet.

The patient was discharged with the advice to continue with oral iron supplements, to introduce 250 mL of soja-based infant milk in the morning and 250 mL in the evening. It was also advice to add an energy powder containing carbohydrates and fat (DUOCAL, Nutricia), (75/kcal/day), to increment the use of legumes, and to eat fish three times a week. We recommended an alimentary diary and continue the checks for failure to thrive (anti-gliadin deamidated antibodies IgG, PCR, VES, TSH, FT4, renal function, blood electrolytes, vitamins A, D, E, K, plasma albumin, LDH, urinary sodium/creatinine and albumin/creatinine ratios, steatocrit, stool occult blood, fecal calprotectin and fecal antigen research for Helycobacter Pylori). Since the parents were also not in favor of the consumption of soja, we recommended the parents replace soya milk with a rice-based infant drink (NovalacRiz, Menarini). An appointment was scheduled after 15 days. The patient did not show up on the day of the appointment and was contacted by phone. The mother reported that they had completed the alimentary diary and partially carried out the recommended laboratory tests (pending results), and also that they have yet not started with the suggested diet. We scheduled a follow-up appointment for the following week however the patient did not attend and was therefore lost to our follow-up.

## 3. Discussion

People following alternative regimes (vegan or vegetarian) are gradually increasing. In particular, the number of vegans has increased by 350% in the past decade [3]. In Europe there are several countries where the percentage of vegetarians reaches 10% of the population (e.g., Germany, Austria and Sweden). In Italy, recent data indicate that vegetarians and vegans have been rising from 6% in 2013 up to 10% in 2016, of whom 1% claimed to be vegan [7,8]. Little information is available on the use of vegetarian and vegan diets in infants and children [9]. North American organization reports 1% prevalence of veganism in children [10]. A recent review conducted on 360 Italian families shows that 8.6% of mothers followed alternative feeding regimen and 9.2% of infants were weaned according to a vegetarian or vegan diet [11]. The pediatrician’s knowledge of these regimens becomes a matter of urgency because, if these diets are not followed in a controlled manner, they will not provide all of the micronutrient requirements, exposing children to nutritional deficiencies, and possible serious health consequences. Furthermore, the earlier they switch to alternative weaning regimes, the more dangerous it can be replacing breastmilk or infant formula with a nondairy drink potentially exposes infants to severe nutritional complications [7,12]. A French study shows that weaning with nondairy drinks is associated with height and weight impairment, neurological complications, electrolyte disturbances, spontaneous bone fracture with bone demineralization and anemia [7]. In the clinical case reported, exclusive breastfeeding was prolonged, a practice that is frequent in mothers who choose alternative regimens [11]. However, the transition to an alternative regimen (which cannot be defined as vegan or vegetarian since the patient was eating fish in small quantities) occurred rather early (10 months of life). According to literature [7], it is possible that this was the cause of anemia and weight impairment. Nonetheless, it is difficult to conclude from a diagnostic point of view because the patient is a celiac potential (familiarity and genetic predisposition), and this remains an important differential diagnosis to be investigated. A fortiori, however, correcting the child’s feeding in a timely and balanced manner could avoid any invasive tests such as esophagogastroduodenoscopy in case of anemia resolution and satisfactory weight gain.

In this context it is legitimate to ask whether this type of diet restriction could be considered a form of child abuse [13,14]. Moreover it is interesting to note that the mothers with psychopathology maternal eating disorders have a tendency to exert excessive control over the diet of their child [15].

What is evident in the reported case is that the parents did not perceive the pediatrician as a reference figure (they had already turned to three pediatricians and were lost to our follow-up). As demonstrated in the study conducted by Baldassarre et al. out of 360 Italian families, there is often an attitude of distrust from parents who choose alternative regimes disregarding the pediatrician. In 22% of cases, in fact, families decide to undertake an alternative regimen without pediatric consultation. Furthermore, in about half cases, families do not consider the pediatrician capable of giving adequate indications regarding the unconventional weaning and find an attitude of opposition from the pediatrician in 77% of cases.

Internationally, pediatric study groups take different positions regarding alternative diets in infants and children. The French-speaking Pediatric Hepatology, Gastroenterology and Nutrition Group (GFHGNP), for example, does not recommend a vegan diet in children. In the event that this is followed, regular dietary monitoring is essential, vitamin B12 and vitamin D supplementation is always necessary, while iron, calcium, docosahexaenoic acid, and zinc should be supplemented on a case-by-case basis [16].The American Academy of Pediatrics and the German Nutrition Society advise against a vegan diet in children [17,18].The Nutrition Committee of European Society for Pediatric Gastroenterology, Hepatology and Nutrition (ESPGHAN) does not recommend this type of diet, but if families wish so, a dietary and medical frame work with regular monitoring should be provided [6]. The Italian Nutrition Society does not contraindicate this type of diet if dietary and medical care is provided [19].

Several working groups (e.g., the Scientific Society for Vegetarian Nutrition—SSNV) are working to provide practical tools for healthcare providers [20] to allow a balanced vegan diet (e.g., by providing oral supplementation scheme for vitamin B12 and vitamin D). In 2017, the SSNV also created the Veg Family Network [21] where experts in the field of vegan nutrition for mothers and children in Italy are listed, so that vegan families can find skilled healthcare professionals for advice.

## 4. Conclusions

The growing number of families who choose an alternative regimen for their child makes the knowledge of these regimens a prerogative of modern pediatricians. The pediatrician should become confident with these types of regimens to make them optimal, adequately inform parents of any risks, and win the trust of such patients. The support from a pediatrician or a nutritional expert for children that follow a vegetarian diet is suitable and recommended. The topic of vegan regimes in the pediatric field remains a delicate one and continues to be discouraged given the serious nutritional deficits demonstrated. However, the difficult management of these selective diets and the mistrust of the parents should justify a continuous updating of a multi-disciplinary approach (pediatrician, psychologist, nutritionist, social worker), and close follow-ups so as to ensure an optimal development of the children.
